# Epigenetic Discrimination: Emerging Applications of Epigenetics Pointing to the Limitations of Policies Against Genetic Discrimination

**DOI:** 10.3389/fgene.2018.00202

**Published:** 2018-06-08

**Authors:** Charles Dupras, Lingqiao Song, Katie M. Saulnier, Yann Joly

**Affiliations:** Centre of Genomics and Policy, McGill University and Genome Quebec Innovation Centre, Montreal, QC, Canada

**Keywords:** epigenetics, DNA methylation, discrimination, insurance, forensic science, ethics, justice, policy

## Abstract

Over more than two decades, various policies have been adopted worldwide to restrict the use of individual genetic information for non-medical reasons by third parties and prevent ‘genetic discrimination’. In this paper, we bring attention to the growing interest for individual *epigenetic information* by insurers and forensic scientists. We question whether such interest could lead to ‘epigenetic discrimination’ – the differential adverse treatment or abusive profiling of individuals or groups based on their actual or presumed epigenetic characteristics – and argue that we might already be facing the limitations of recently adopted normative approaches against genetic discrimination. First, we highlight some similarities and differences between genetic and epigenetic modifications, and stress potential challenges to regulating epigenetic discrimination. Second, we argue that most existing normative approaches against genetic discrimination fall short in providing oversight into the field of epigenetics. We conclude with a call for discussion on the issue, and the development of comprehensive and forward-looking preventive strategies against epigenetic discrimination.

## Introduction

Epigenetics has been defined as “the study of changes in gene function that are mitotically and/or meiotically heritable and that do not entail a change in DNA sequence" (Wu and Morris, 2001, 1104). By assessing DNA methylation levels and/or histone modifications in specific cell types, for instance, epigenetic tests may soon provide additional layers of predictive information, complementary to genetic information, about an individual’s disease risk profile or response to specific treatments. They could also provide information about someone’s past exposures to physico-chemical (e.g., toxic pollutants, cosmetics) and psychosocial (e.g., familial stress, social adversity) disruptors of epigenetic mechanisms. These emerging opportunities have recently generated concerns, legal scholars and bioethicists, regarding the level of protection for patients’ and research participants’ privacy and confidentiality in epigenetics. According to some authors, the degree to which epigenetic databases are secured, and their access appropriately restricted to accredited users, should be carefully considered (Philibert et al., 2014; Diemer and Woghiren, 2015; Dyke et al., 2015; Backes et al., 2016). Indeed, it is to some extent unclear how well existing regulatory mechanisms and encryption algorithms developed for genetic information are suited for the protection of epigenetic information (Terry, 2015).

In this paper, we bring attention to an additional set of ethical concerns, related to the use of epigenetic information by third parties for discriminatory purposes (i.e., abusive profiling or adverse differential treatment) that would negatively impact the lives of patients, research participants or other vulnerable groups. We present and discuss some anticipated and emerging applications of epigenetics that could lead to *epigenetic discrimination*. So far, the literature on the matter has been scarce and mostly speculative (Rothstein et al., 2009; Katz, 2013; Juengst et al., 2014; Mansfield and Guthman, 2015; Saulnier and Dupras, 2017). Although the value of epigenetic data for providing health or uniquely identifying information is still to be determined, the rapidly developing interest of the insurance industry and forensic experts in accessing individual epigenetic information is, we argue, calling for closer ethical scrutiny.

## Genetic Discrimination and the Exceptionalism Position

Over the past decades, a few high-profile incidents of *genetic discrimination* have created a significant amount of public concern. Controversies have arisen regarding the moral and legal acceptability of using individuals’ genetic information to exclude some people from accessing a variety of social goods (Hudson et al., 1995; Lemmens, 2000; Hellman, 2003; Otlowski et al., 2012; Joly et al., 2013). Discussions about genetic discrimination usually refer to discriminatory practices by insurance companies or by employers, who would be tempted to require that individuals undergo or disclose the results of a genetic test (Rothstein, 2008; Joly et al., 2013).

To address this problem, countries all over the world have adopted a diversity of public policies (see Joly et al., 2017b). For example, in the United States, the *Genetic Information Non-discrimination Act* (GINA) was enacted to provide greater protection to asymptomatic individuals against denial of health insurance or employment based on a genetic predisposition to diseases. In May 2017, Canada passed into law a similar legislative framework, the *Genetic Non-Discrimination Act* (GNA), to prevent any person – except for health care practitioners and researchers (OpenParliament.ca, 2017) – from requiring an individual to undergo a genetic test or disclose the results of a genetic test as a condition for entering into or continuing a contract or agreement for providing goods and services (GNA, 2017).

‘Genetic exceptionalism’, i.e., the largely shared presumption that genetic information is ethically sensitive to the point where it deserves special attention and treatment in our laws and policies, has served as a basis to justify the majority of non-genetic discrimination public policies that have been enacted worldwide. The exceptionalism view, however, has been criticized by many scholars over the past decades (Gostin and Hodge, 1999; Suter, 2001; Green and Botkin, 2003; Hellman, 2003; Rothstein, 2005, 2013; Lemke, 2005). Indeed, it is not always clear what intrinsic properties of the DNA molecule (e.g., DNA sequence, genetic mutation) make it more deserving of protection than other types of information contained in the medical record of an asymptomatic, at-risk person (e.g., familial history of disease, cholesterol level and high blood pressure).

## Potential Discriminatory Uses of Epigenetic Information

In November 2016, GWG Holdings’ insurtech subsidiary Life Epigenetics secured an exclusive license over the exploitation of a new epigenetic technology allegedly allowing for the prediction of a person’s life expectancy through DNA methylation profiling. Originally developed by UCLA researcher Steve Horvath, the technique had been patented (PCT/US2014/058089) a year before by The Regents of the University of California (Horvath, 2015; Genomeweb, 2017). In March 2017, the insurance company GWG Life announced that it had already started collecting and analyzing samples of saliva provided by their policy owners in order to determine their true ‘biological age’, building on individual epigenetic information (GWG-Holdings-Inc., 2017).

The collection by GWG Life of saliva samples from life insurance policy owners, for subsequent analysis of DNA methylation levels, suggests the possibility that some insurers might stratify their clients based on their epigenetic information. The deceptive commercial promotion of this approach is concerning. Most notably, we found a recent report of consumer opinion by GWG Holdings especially misleading; not only for having been extrapolated from a study conducted with insurance agents and financial advisors, but also for a very confusing graphic presentation of the results of their survey in Nasdaq Globenewswire (independent variables ‘likely’ and ‘very highly’ being favorably inverted on the vertical axis) (GWG-Holdings-Inc., 2018).

An important question also arises: how fair is it to engage in differential treatment based on people’s epigenetic profile? According to GWG Life, those proving epigenetically younger than their ‘chronological age’ should be rewarded with premium life insurance underwritings (i.e., access to insurance at a lower price) (Karow, 2009). According to the actuarial logic deployed as a justification – and which insurance companies have consistently referred to in the context of genomics – accounting for ‘actuarial fairness’ requires full disclosure of all relevant medical information by insurance applicants (Knoppers and Joly, 2004). In failing to do so, an applicant would be ‘cheating’ other members of the insurance pool, and most importantly, those of lower risk groups. However, it is important to note that the argument of actuarial equity does not stand up to ethical scrutiny by other important theories of justice (e.g., desert-based or luck-egalitarian accounts) that would prohibit the disadvantageous differential treatment of some individuals or groups based on dispositions that are not within their control, but are a matter of brute luck (see Silvers and Stein, 2002). Concerns have also arisen with the probability that genetic discrimination may disproportionally burden individuals belonging to already vulnerable groups (e.g., targeting African Americans because they have a higher genetic risk of developing sickle cell anemia, or immigrants seeking to reunite with family members in their new country of adoption) (see Eltis, 2007; Joly et al., 2017c).

The latter two objections have been instrumental in the development and implementation of various normative approaches against genetic discrimination over the past three decades. It is highly probable, we argue, that the distinction between those biological traits that are perceived as inherited (not subject to control) vs. those acquired throughout life (some possibly subject to some degree of control) will re-emerge in discussions about epigenetic discrimination. This is likely to occur considering recent publications pointing to the detrimental effects of a *bad* lifestyle (e.g., at-risk dieting, smoking, alcohol consumption), which gets ‘embedded’ in the individual’s genes through epigenetic mechanisms, and may be detected later in life using epigenetic tests (Haycock, 2009; Anderson et al., 2012; Knopik et al., 2012; Breitling, 2013; Lee and Pausova, 2013; Nieratschker et al., 2014; Mahnke et al., 2017).

Questionable potential and emerging applications of epigenetics research also include other types of differential treatment and abusive profiling that have been more neglected by scholars in the past, namely in activities falling under governmental mandates such as immigration control and forensic investigations (Joly et al., 2017b; Shabani et al., 2018). These activities deserve closer scrutiny, considering that some governments have recently allowed increasingly permissive genetic profiling strategies (for ex. familial searches and DNA dragnet) that are at odds with individual liberties and ethical principles, such as the rights to privacy and social inclusion (CCLA, 2016; Granados Moreno et al., 2017; Joly et al., 2017c). It should also be noted that minority population groups such as Native Americans and African Americans, while underrepresented in health research genomic databases, are unfairly overrepresented in DNA criminal databases in comparison to Caucasians due to the over-policing of these populations (MacAttram, 2009; Chow-White and Duster, 2011).

The use of *genetic* information in the field of forensics has also been criticized for having led to some unjustified criminal convictions based on misapplied or faulty science (NRC, 2009). At the same time, there is growing interest in the use of *epigenetic* testing in forensic labs to generate evidence that could not be uncovered using genetic testing, including information about the approximate age of a subject and identification of the source of biological fluid samples (Vidaki and Kayser, 2017). Other researchers have expressed concerns about the possible use of epigenetic data to identify biomarkers of psychopathic neurotypes that might be used to detect a perceived risk of future criminality in individuals who have not yet committed any crimes. Different interpretations of science and/or public interventions grounded on statistical associations between such epigenetic risks and affiliation to specific socio-cultural communities might also contribute to increasing their stigmatization (Mansfield, 2012). Thus, overly deterministic readings of epigenetic marks could promote discriminatory attitudes, discourses and practices based on the predictive nature of epigenetic information. Interestingly, non-deterministic readings of epigenetics could also promote stigmatization and discrimination, this time based on the presumed capacity of individuals to avoid harmful exposures during their lifetime (Meloni, 2017). It is, however, important to remain cautious about the interpretation that epigenetic modifications, in contrast with genetic mutations, are the result of free and voluntary decisions on the part of at-risk individuals (Chiapperino and Testa, 2016; Dupras and Ravitsky, 2016b; Vears and D’Abramo, 2017).

## Conceptual Challenges to Defining Epigenetic Discrimination

For the moment, it is impossible to ascertain that DNA methylation levels such as those currently being tested by GWG Life have been acquired during the life course. They may for instance have been acquired before birth, or even prior to conception. In addition, many scientists agree that a very large proportion of inter-individual epigenetic variability is simply a *consequence* of inter-individual genetic variability. According to them, most epigenetic modifications are ‘obligatory’, or minimally, ‘facilitated’ by an individual’s genes (see Whitelaw and Whitelaw, 2006; Heard and Martienssen, 2014). From that perspective, someone’s epigenetic profile appears more as a downstream result of innate biological predispositions, rather than acquired disruptions (Levine et al., 2018).

Important tensions thus arise in discussions about epigenetic discrimination, and with regards to the long-lasting debate about genetic exceptionalism. A first challenge lies, we argue, in the underestimated *blurred frontier* between what should be considered ‘genetic’ and what should be considered ‘epigenetic’. Indeed, it is not clear at all whether DNA methylation shares more biological properties with histone modifications (highly dynamic, potentially reversible), or with genetic mutations (stable in time, potentially inherited). We should be cautious with simplistic, most often dichotomous conceptualizations of ‘genetics’ and ‘epigenetics’. Reflecting on the possible underlying reasons for the usual opposition of these fields can be quite instructive. As aptly observed by Lappé and Landecker (2015), it may lead us to challenge the most often used definition of ‘epigenetics’ that we presented at the very outset of this paper, and which explicitly excludes changes to the DNA sequence. According to them, DNA methylation could in fact be perceived as changing DNA’s linear nucleoid sequence when transforming cytosines (C) into methylcytosines (meC). Following this logic, the line between genetics and epigenetics appears much more blurred than what is usually admitted; so too could be the line between genetic and epigenetic discrimination.

In addition to the difficulty of delineating the frontier between epigenetics and genetics there exists perhaps an even greater challenge: that of handling the potential normative implications of the *high heterogeneity* of the field of epigenetics. As argued elsewhere, the diversity of epigenetic mechanisms and variants – and different contexts under which epigenetic programming occurs – can impact the attribution of moral epigenetic responsibilities to different actors (Dupras and Ravitsky, 2016a). Ambiguities related to ‘epigenetic plasticity’ – arising from apparent contradictions between the *dynamism/reversibility* of some epigenetic variants and the long-term *stability/inheritance* of others – and ‘epigenetic normality’ – arising with the difficulty of distinguishing *epigenetic disruption/harm* from *epigenetic programming/adaptation* – may also be relevant to discussions about epigenetic discrimination. These two ambiguities are important to consider, we argue, since they introduce significant conceptual challenges into the identification of, respectively, epigenetic variants over which individuals may be able to exercise control (but also to what extent, to whom and when), and epigenetic variants that should be considered at-risk and/or healthy.

It is not easy to determine whether DNA methylation and histone modifications, for instance, are conceptually equivalent and deserve equal protection against discrimination. In fact, the biological properties of methylated cytosins are considerably distinct from other epigenetic modifications, such as histone acetylation or histone phosphorylation. DNA methylation involves the creation of a relatively stable chemical bond between a methyl group and a nucleic acid (covalent bond). Histone modifications, in contrast, imply much weaker chemical bonds between histone tails and the DNA (hydrogen bonding). For that reason, the latter often last only for a few hours following a stimulus, whereas DNA methylation can be stable for many years, persist throughout life, and sometimes even be passed on to future generations (Jenuwein and Allis, 2001). Considering the lack of homogeneity among diverse types of epigenetic mechanisms and variants, it is unclear how one could imagine oversight applying similarly to all types of epigenetic information.

## Current Non-Discrimination Policies Falling Short for Oversight

In a previous study, we critically reviewed the different normative approaches to genetic discrimination worldwide (Joly et al., 2017b). We reported and characterized eight significantly distinct approaches: human rights, genetic exceptionalism, sectoral prohibition, ethical guidance, self-regulation, moratoria, status-quo and hybrids. We also highlighted their individual advantages and limitations. Using the same database (see ‘supplemental information’ in Joly et al., 2017b), two investigators (CD and LS) later searched the content of definitions privileged by each country for any mention of the keywords “epigenetics,” “DNA methylation,” or “histone modification”. The aim was to identify possible allusions to discrimination based on epigenetic testing or information, and appeals for these policies to apply (or not) to epigenetics. We found that none of the existing genetic anti-/non-discrimination policies *explicitly* include epigenetic testing or information within their terminology and scope. While the content of some definitions may *implicitly* include some epigenetic components, policy-makers generally do not seem to have considered the potential implications of this emerging discipline. Instead, they privileged narrowly framed, short-sighted approaches regulating only the use of genetic components – and specifically when revealed through genetic testing.

A tangible explanation for the absence of explicit mentions of epigenetics in existing policies is the relative novelty of the field of epigenetics, and the fact that legislation often lags well behind scientific development (Rugg-Gunn et al., 2009). In many European countries, for instance, genetic non-discrimination policies rely on the following provision, issued first in 1997 by the Council of Europe as Article 12 of the *Convention on Human Rights and Biomedicine* (Council of Europe, 1997): “Tests which are predictive of *genetic diseases* or which serve either to identify the subject as a *carrier of a gene* responsible for a disease or to detect a *genetic predisposition or susceptibility* to a disease may be performed only for health purposes or for scientific research linked to health purposes, and subject to appropriate *genetic counseling*” (our italics). The focus of this provision on the ‘gene’ is compelling. Normative approaches that are predicated solely on such statements would most likely not apply to epigenetic testing and information (Rothstein, 2013).

## Conclusion

In addition to the two arguments mentioned above (i.e., individuals’ lack of control over the genes they inherit and need to protect already vulnerable groups), an underlying objective of non-discrimination policies is to prevent the potential adverse consequences of public concern of genetic discrimination. Emerging evidence that such fears will affect patients’ adherence to physicians’ recommendations to undergo genetic testing, or recruitment of participants into genetics research, has indeed served as an additional justification for oversight (Joly et al., 2013, 2017a; Bombard and Heim-Myers, 2018). Considering the probable rise of similar issues in the context of epigenetics, but also with the rapid development of proteomics, metabolomics, microbiomics, mobile health applications and big data analytics, we call on the bioethics community for a comprehensive debate on epigenetic discrimination, and other potential forms of discriminatory practice based on new types of biological data. An interdisciplinary appraisal of this matter is timely and necessary to guide the development of efficient strategies against potential misuses of epigenetic information.

Further investigation about the scientific and clinical validity of the technology that is being used by the industry is also required. What are the rates of false positives and false negatives of an epigenetic test? Has it been validated for all types of populations, or is its predictive accuracy limited to specific groups? Does it meet well-established professional and regulatory standards? And how are testing protocols planning to account for possible variations in a person’s epigenetic profile during their lifetime? In the case of GWG Life’s new test, for instance, is the correlation between someone’s gross DNA methylation profile (i.e., not gene-specific), biological age, and life expectancy sufficiently strong to justify a different treatment in actuarial terms? Finally, it will be important to determine whether and under what circumstances it is morally acceptable to discriminate against people using epigenetic information about an individual’s life expectancy, that has been associated neither to a specific disease, nor to a specific causal factor of epigenetic variability among persons.

## Author Contributions

CD wrote the first draft of the paper. LS, KS, and YJ contributed significantly by improving the arguments, language, flow, and overall structure of the paper.

## Conflict of Interest Statement

The authors declare that the research was conducted in the absence of any commercial or financial relationships that could be construed as a potential conflict of interest.
